# Permian ginkgophyte fossils from the Dolomites resemble extant *O-ha-tsuki *aberrant leaf-like fructifications of *Ginkgo biloba *L

**DOI:** 10.1186/1471-2148-10-337

**Published:** 2010-11-03

**Authors:** Thilo C Fischer, Barbara Meller, Evelyn Kustatscher, Rainer Butzmann

**Affiliations:** 1 Department Biology I, Biocenter Botany, Ludwig-Maximilians-University Munich, Großhadernerstrasse 2-4, D-82152 Planegg-Martinsried, Germany; 2 Institute of Palaeontology, Geocenter, University Vienna, Althanstrasse 14, A-1090 Wien, Austria; 3 Museum of Nature South Tyrol, I-39100 Bozen/Bolzano, Bindergasse 1, Italy; 4 Fuggerstrasse 8, D-81373 München, Germany

## Abstract

**Background:**

Structural elucidation and analysis of fructifications of plants is fundamental for understanding their evolution. In case of *Ginkgo biloba*, attention was drawn by Fujii in 1896 to aberrant fructifications of *Ginkgo biloba *whose seeds are attached to leaves, called *O-ha-tsuki *in Japan. This well-known phenomenon was now interpreted by Fujii as being homologous to ancestral sporophylls. The common fructification of *Ginkgo biloba *consists of 1-2 (rarely more) ovules on a dichotomously divided stalk, the ovules on top of short stalklets, with collars supporting the ovules. There is essentially no disagreement that either the whole stalk with its stalklets, collars and ovules is homologous to a sporophyll, or, alternatively, just one stalklet, collar and ovule each correspond to a sporophyll. For the transition of an ancestral sporophyll resembling extant *O-ha-tsuki *aberrant leaves into the common fructification with stalklet/collar/ovule, evolutionary reduction of the leaf lamina of such ancestral sporophylls has to be assumed. Furthermore, such ancestral sporophylls would be expected in the fossil record of ginkgophytes.

**Results:**

From the Upper Permian of the Bletterbach gorge (Dolomites, South Tyrol, Italy) ginkgophyte leaves of the genus *Sphenobaiera *were discovered. Among several specimens, one shows putatively attached seeds, while other specimens, depending on their state of preservation, show seeds in positions strongly suggesting such attachment. Morphology and results of a cuticular analysis are in agreement with an affiliation of the fossil to the ginkgophytes and the cuticle of the seed is comparable to that of Triassic and Jurassic ones and to those of extant *Ginkgo biloba*. The *Sphenobaiera *leaves with putatively attached seeds closely resemble seed-bearing *O-ha-tsuki *leaves of extant *Ginkgo biloba*. This leads to the hypothesis that, at least for some groups of ginkgophytes represented by extant *Ginkgo biloba*, such sporophylls represent the ancestral state of fructifications.

**Conclusions:**

Some evidence is provided for the existence of ancestral laminar ginkgophyte sporophylls. Homology of the newly found fossil ginkgophyte fructifications with the aberrant *O-ha-tsuki *fructifications of *Ginkgo biloba *is proposed. This would support the interpretation of the apical part of the common *Ginkgo biloba *fructification (stalklet/collar/ovule) as a sporophyll with reduced leaf lamina.

## Background

**Figure 1:**
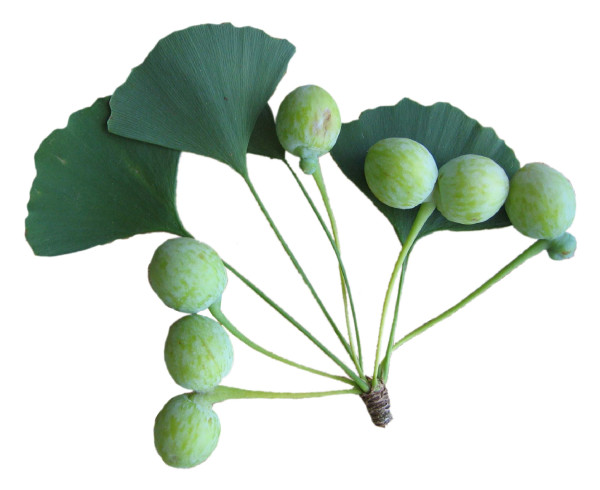
***Fertile shoot of female *Ginkgo biloba**. The common female fructification of *Ginkgo biloba *is a brachyblast (spur shoot) with sterile leaves (trophophylls) and ovules on stalks, the latter either being interpreted as structures derived from sporophylls, or as shoots, or as organs composed of both.

**Figure 2:**
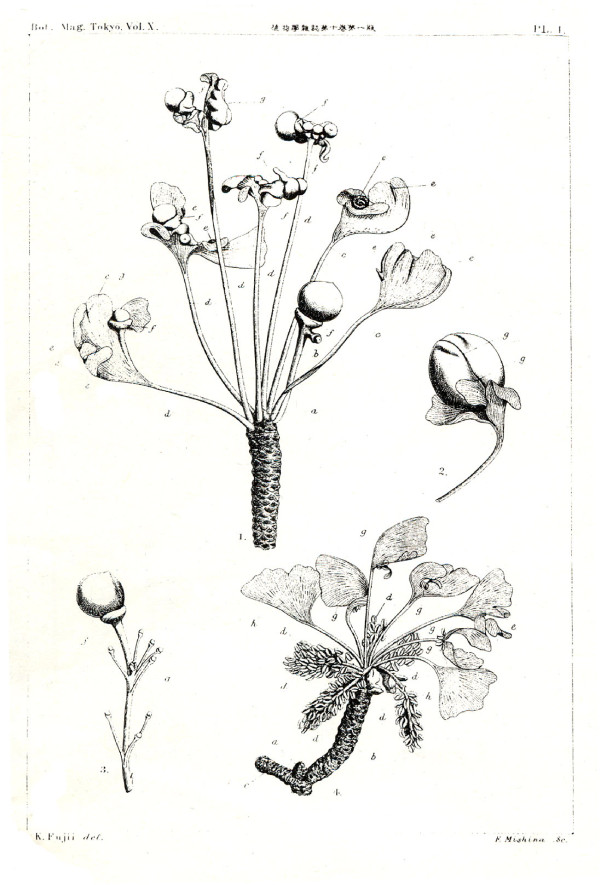
***Historical figure by Fujii (1896) on the *O-ha-tsuki *phenomenon of *Ginkgo biloba**. Plate from Fujii (1896) demonstrating rare female *O-ha-tsuki *("seed at leaf") type aberration of *Ginkgo biloba *fructifications (1-1: whole fertile brachyblast, 1-2: single ovule at leaf, its male analogue (1-4), and a rare multi-ovulate fructification with a bud (1-3)).

**Figure 3:**
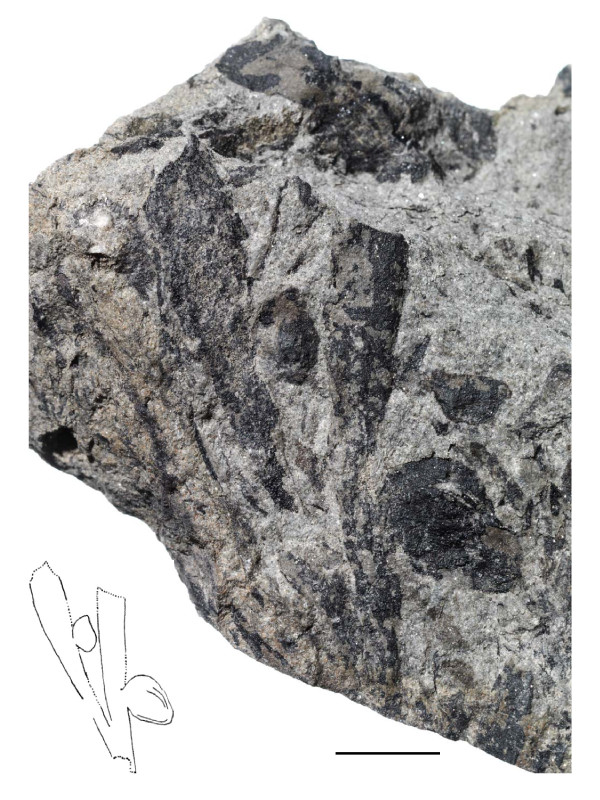
**Ginkgophyte leaf with putatively attached seeds from the Upper Permian of the Bletterbach (Dolomites)**. Morphology of the specimen (inventory number PAL-1368), drawing of it performed with binocular control. Evident structures drawn as lines, supposed structural outline as dotted lines. Scale bar 10 mm.

In 1869 Van Tieghem [1] provided a first interpretation of the female organs of *Ginkgo biloba *(figure 1) in his work on comparative anatomy of fructifications. In his view the whole stalk, cupule and ovule are homologous to a sporophyll. Later (1896), Fujii [2] provided a scientific description of the long-known *Ginkgo biloba O-ha-tsuki *leaves with seeds (figure 2-1, 2-2: female, 2-4: male counterpart). Based on the rare presence of an axillary bud in multi-ovulate forms of the fructification (figure 2-3), the basal part of the stalk was interpreted by him as being shoot-derived. The stalklet, collar and ovule would correspond to petiole, reduced lamina and ovule of a sporophyll. Other opinions, mainly the interpretation as a two-flowered inflorescence (Strasburger), are also summarized and cited in Fujii [2]. Wettstein [3], Sakisaka [4] and others (cited therein) essentially agreed with Fujii's view and extended the argumentation by considering also the vascularisation of the organs. Vegetative leaves of *Ginkgo biloba *have two vascular bundles at the petiole base, whereas the stalk of the common fructification with two ovules has four (or correspondingly more in the rare forms with multiple ovules). *O-ha-tsuki *leaves possess two bundles, like vegetative leaves. This provides further evidence to homologise them only with the stalklet/collar/ovule - distal part of the common *Ginkgo biloba *fructification. Van Tieghem's view, in contrast, that the whole female organs are homologous to a sporophyll could be supported by interpreting the rare axillary bud as an ectopic development of a meristem, but the vascular system of the stalk with four bundles could hardly be explained. However, the *O-ha-tsuki *form was also interpreted only as a case of retroconvergent morphology, arguing with the absence of fossil occurrences of "leafy ovuliphores" in the Mesozoic [5].

Fossil ginkgophyte fructifications are rare in the fossil record [6]. Most closely related to *Ginkgo biloba *are ovulate structures attached to brachyblasts (spur shoots) carrying also *Ginkgo*-like vegetative leaves (or associations of both in the fossiliferous sediments) from the Mesozoic and Cenozoic: *Ginkgo yimaensis *(Jurassic) [7], *Ginkgo apodes *(Late Jurassic to Early Cretaceous) [8], and *Ginkgo adiantoides *leaves/*Ginkgo geissertii *ovule (Cenozoic) [9,10]. *Nehvizdyella bipartita *(Late Cretaceous) [11] has similar fructifications, but non-divided leaves. The fossil taxa affiliated to *Ginkgo *can be ordered in an evolutionary series with successive reduction of individual stalks, number of ovules and accompanying increase in the size of ovules and in the width of the leaf segments, reflecting also ontogenetic sequences in *Ginkgo biloba *[12]. Palaeozoic fructifications of ginkgophytes are also known, but much more difficult to interpret. *Trichopitys heteromorpha *from the Lower Permian [13] is the oldest generally accepted ginkgophyte [[14], and other authors]. It consists of axes carrying dichotomous, non-laminar leaves with zones of dividing stalks with terminal ovules which are possibly recurved. *Karkenia *is a genus with multi-ovulate axes or multi-ovulate globular fructifications; the first species (associated with *Ginkgoites tigrensis *leaves) was described by Archangelsky from the Lower Cretaceous of Patagonia [15]. Another well known member is the Liassic *Karkenia hauptmannii*, represented by brachyblasts with globular fructifications and *Sphenobaiera *leaves [16]. *Avatia bifurcata *and *Hamshawvia longipedunculata *from the Triassic of South Africa [17] also possess globular multi-ovulate fructifications (on dichotomous stalks). *Hamshawvia *fructifications are found attached to brachyblasts with *Sphenobaiera *vegetative leaves.

Recently, Naugolnykh [18] has reviewed "foliar" seed-bearing organs of Paleozoic ginkgophytes. Some of the taxa he included in the ginkgophytes, e.g., *Arberia*, have been interpreted differently i.e. as glossopterid [14]. Taxa that can be accepted as ginkgophyte seed-bearing organs clearly have a dichotomous structure like *Trichopitys*, *Karkenia*, *Toretzia*, and *Grenana*. These genera show stalks carrying ovules. If accepted as being homologous to leaves (sporophylls), these either would represent non-laminar leaves, or, alternatively, the laminar part would have been already reduced. The latter possibility led Naugolnykh to suppose "pre-karkeniaceous" ancestors of ginkgophytes.

Here we report on Permian laminar ginkgophyte leaves with putatively attached seeds.

## Results

### Description of the fossils from the Upper Permian of the Dolomites and comparison with ginkgophyte characters

#### Morphology

**Figure 4:**
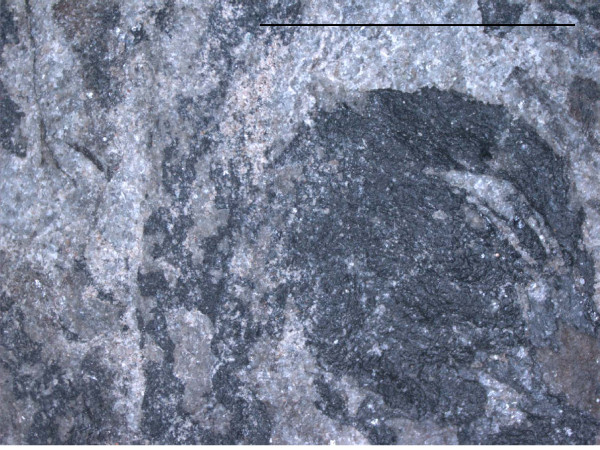
**Ginkgophyte leaf with putatively attached seeds from the Upper Permian of the Bletterbach (Dolomites), detail**. Detail of PAL-1368 at the assumed attachment site of the seed. Scale bar 10 mm.

Numerous specimens of vegetative *Sphenobaiera *leaves (wedge-shaped, without petiole) were discovered from a lens of fossiliferous argillaceous siltstone to fine-grained sandstone at the Upper Permian Bletterbach locality [19]. Among these, some specimens were identified where also seeds are preserved (the described specimens are called "seeds", even if "ovules" is often used for ginkgophyte seeds). Figure 3 and 4 illustrate a specimen of a wedge-shaped, dichotomously divided leaf with all characteristics of the genus *Sphenobaiera*, but with two seemingly attached seeds. Especially in the case of the larger seed, which is in a lateral position at the leaf margin, the organic connection to the leaf segment is assumed. There is no basal collar-like structure in the fossil like at extant *Ginkgo biloba *ovules or like the irregular basal swellings in the *O-ha-tsuki *form. This type of fossil seeds occurred only on some of the approximately one hundred blocks of sediments from the Bletterbach collection with *Sphenobaiera *leaves, in close association in each case to such leaves, and not randomly on other blocks of sediment with various plant fossils. None of this type of seed showed a stalk or remains of such.

#### Cuticular structures

Cuticles were prepared from the leaf lamina region of the counterpart of the first specimen (figure 3).

**Figure 5:**
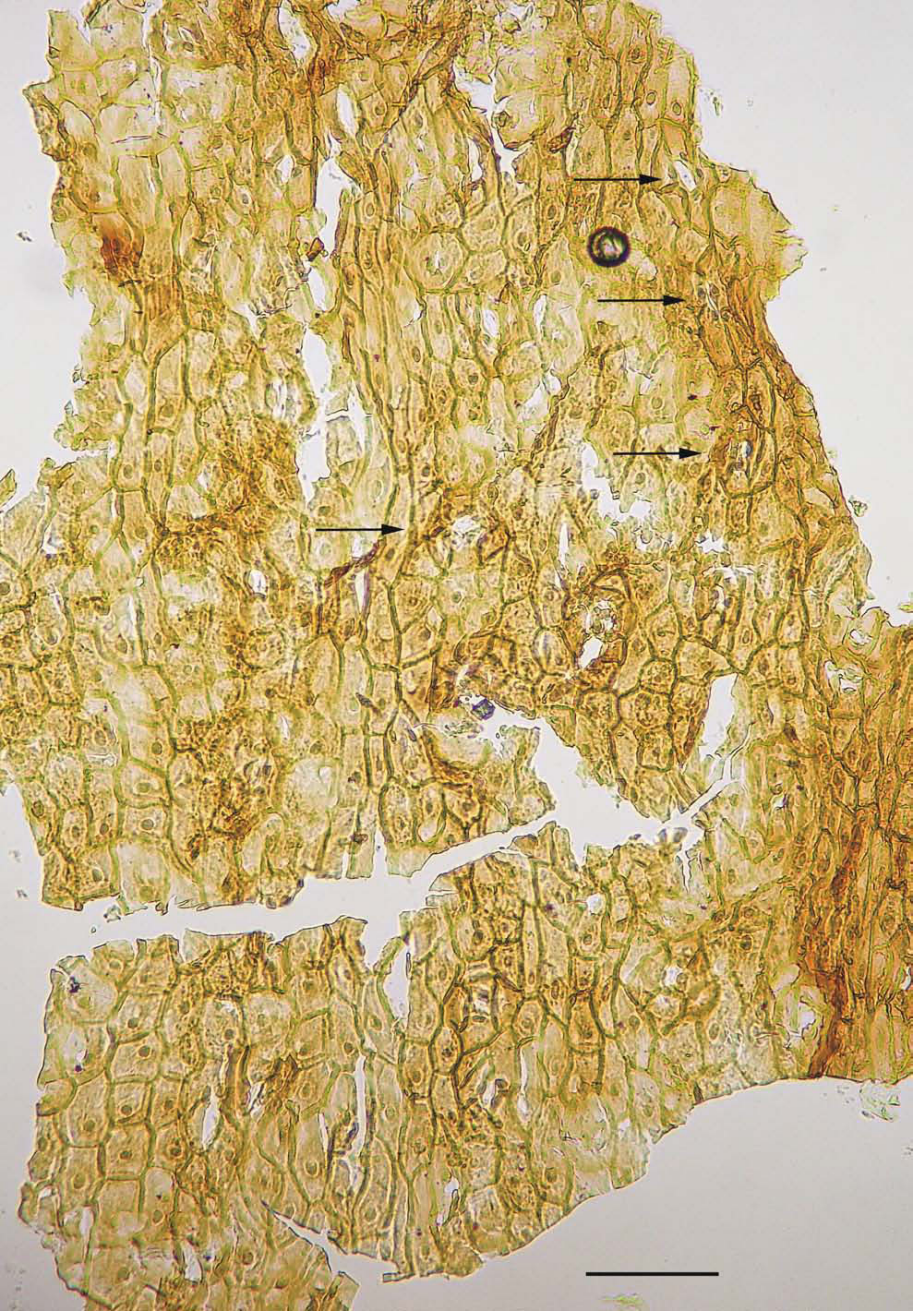
**Abaxial cuticle prepared from the leaf lamina region of ginkgophyte leaf with putatively attached seeds**. Abaxial cuticle prepared from the leaf lamina region of the specimen PAL-1368. The arrows point to stomatal complexes. Scale bar 100 μm.

**Figure 6:**
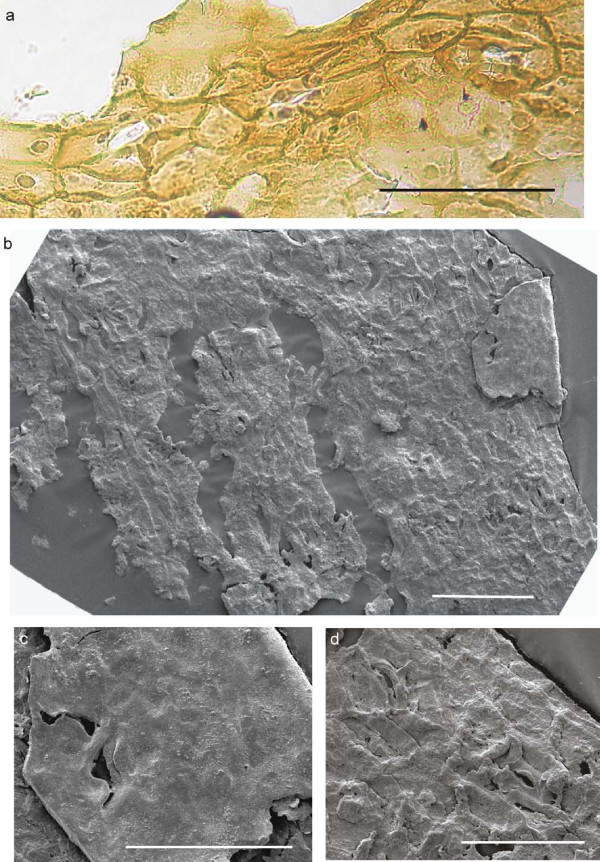
**Details of abaxial cuticles prepared from the leaf lamina region of ginkgophyte leaf with putatively attached seeds**. a: Stomatal complexes of figure 5in detail. Scale bar 100 μm. b: Inner side of the abaxial cuticle of the lamina; at the right margin the surface of the outside is also visible (detail in figure 6 c) (SEM). Scale bar 100 μm. c: The nearly smooth outside of the abaxial leaf cuticle with papillae, indicating a stoma slit. The whitish spots on the surface probably derive from the wax layer (SEM). Scale bar 50 μm. d: Inner side of the abaxial leaf cuticle with two stomata (SEM). Scale bar 50 μm.

**Figure 7:**
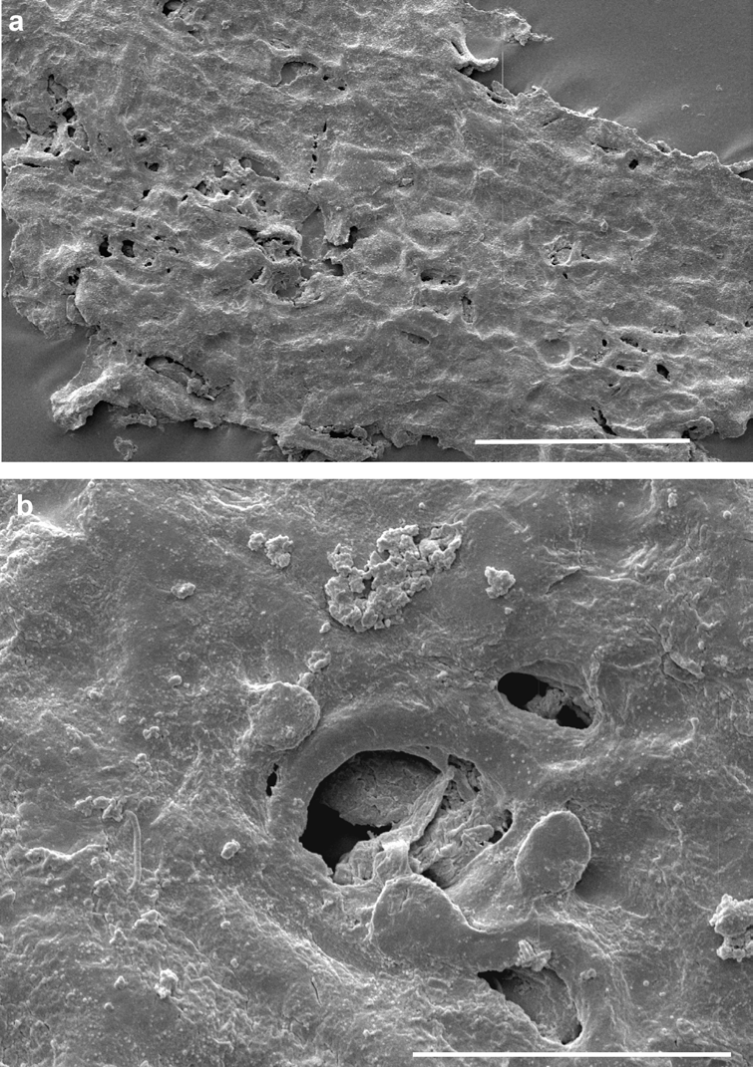
**Details of adaxial cuticles prepared from the leaf lamina region of ginkgophyte leaf with putatively attached seeds**. a: Inner side of adaxial cuticle of the lamina (SEM). Scale bar 50 μm. b: Outside of the adaxial cuticle of the lamina with one stoma, surrounded by papillae (SEM). Scale bar 20 μm.

The abaxial cuticle of the leaf (figure 5) is formed by cells of different shapes and sizes: polygonal, rectangular, isodiametric and elongated ((15-)25-75(-100 μm)) which are partly arranged in irregular rows. One longitudinal band with two rows of elongated cells is visible in the middle of the figure, probably representing the costal area of a vein; the cells bear small papillae. Two other bands at the right and left margin are less distinct but may represent additional costal areas. Anticlinal walls (this is the cell outline on the prepared cuticles) are straight, sometimes slightly curved. Stomatal complexes are not all well preserved. They are arranged in bands (putative intercostal regions) and are surrounded by more polygonal-isodiametric cells. The stomatal complexes are about 50-60 μm in diameter, rarely up to 90 μm. The 4-6 subsidiary cells (figure 5, 6a) have small marginal papillae almost covering the stomatal pit. Guard cells are rarely recognizable. Similar cuticle characteristics have been found for other fossil ginkgophytes [7,9,20] - [23]. Under the scanning electron microscope (SEM), the outside of the abaxial cuticle appears to be nearly smooth (figure 6c). The papillae are visible as flat bulges. The inner side not always distinctly displays the anticlinal walls, but stomata are clearly recognizable (figure 6b, d). The adaxial cuticle of the leaf shows only few preserved rectangular anticlinal walls with the light microscope (LM) (not figured) and indistinct anticlinal walls with the SEM. Also one stomatal complex is visible with the SEM (figure 7a, b).

**Figure 8:**
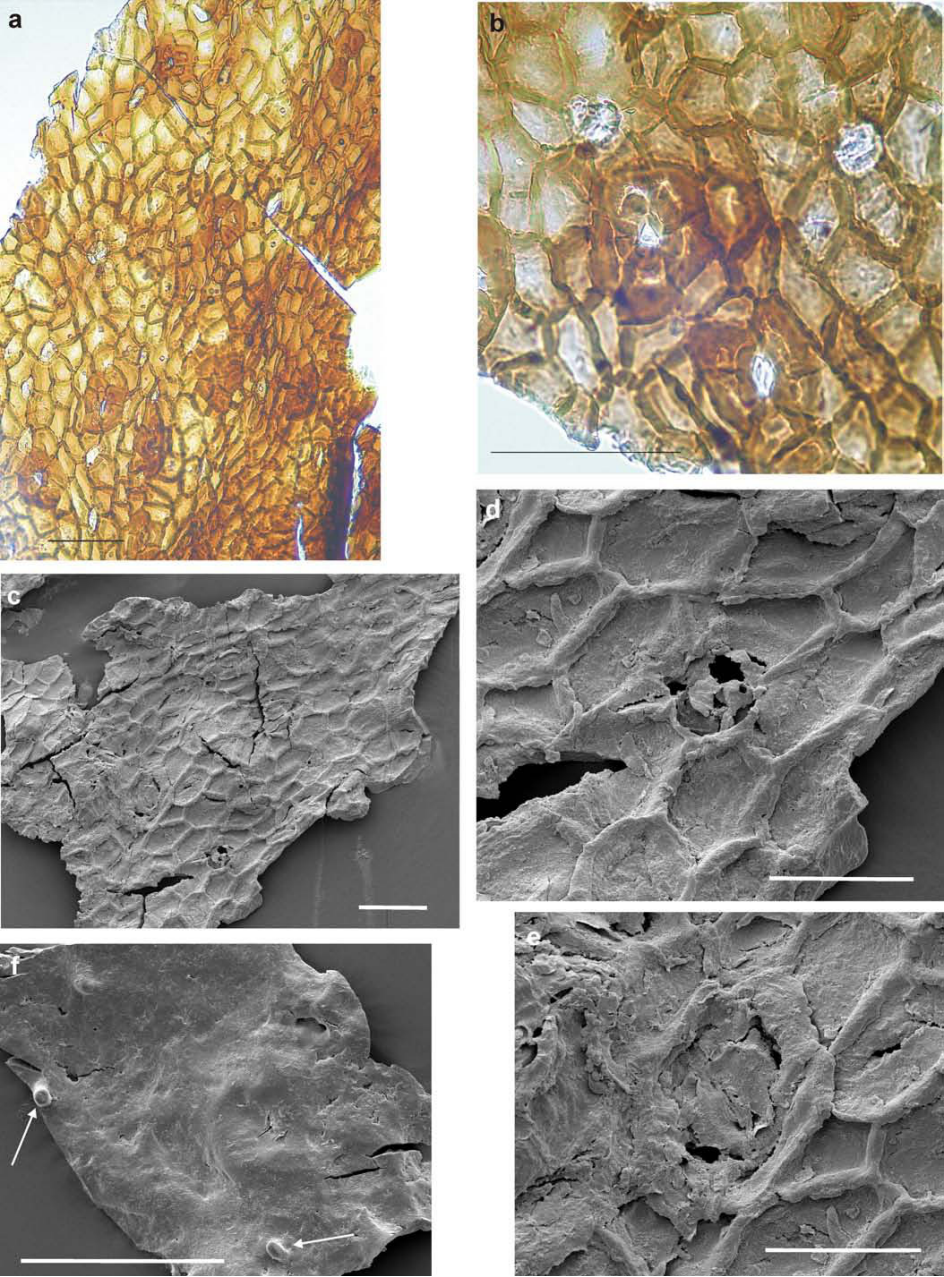
**Details of cuticle prepared from the seed of ginkgophyte leaf with putatively attached seeds**. a: Cuticle prepared from the seed of the specimen with several stomatal complexes and idioblasts. Scale bar 100 μm. b: Seed cuticle with stomatal complexes in detail; the lower one shows also the guard-cells. In the upper part of the picture two idioblasts are shown in detail. Scale bar 100 μm. c: Inner side of the seed cuticle (SEM). Scale bar 100 μm. d: Detail of figure 8 c showing an idioblast (SEM). Scale bar 50 μm. e: Detail of figure 8 c showing one stomatal complex. Scale bar 50 μm. f: Outside of the cuticle with two tubes (arrows) and at least one stomatal complex with very weakly developed papillae. Scale bar 100 μm.

The cuticle of the seed (figure 8a-f) shows small cells with mainly straight, rarely slightly curved anticlinal walls. They are of polygonal shape, irregularly arranged and without papillae. The cell diameter varies from (14-)25 to 55 μm, rarely up to 70 μm. The stomatal complexes with the subsidiary cells are 60 to 95 μm in diameter. The 5-6 subsidiary cells of mainly trapezoid shape (diameters about 14-39 μm) are distinctly thickened at the inner margin reducing, thus, the stomatal pit (figure 8a, b). Details of the guard-cells are rarely visible (figure 8b). Other circular to elliptical pits, about 30-35 μm in diameter, which are also enclosed by six cells but are not thickened, are idioblasts (figure 8d). The shown outside surface of the seed cuticle (figure 8f) is almost smooth with at least one stomatal complex with weakly developed papillae and two tubes, which might derive from the wax layer.

#### Comparison to cuticles of other ginkgophytes

##### Cuticles of extant Ginkgo biloba (O-ha-tsuki form)

**Figure 9:**
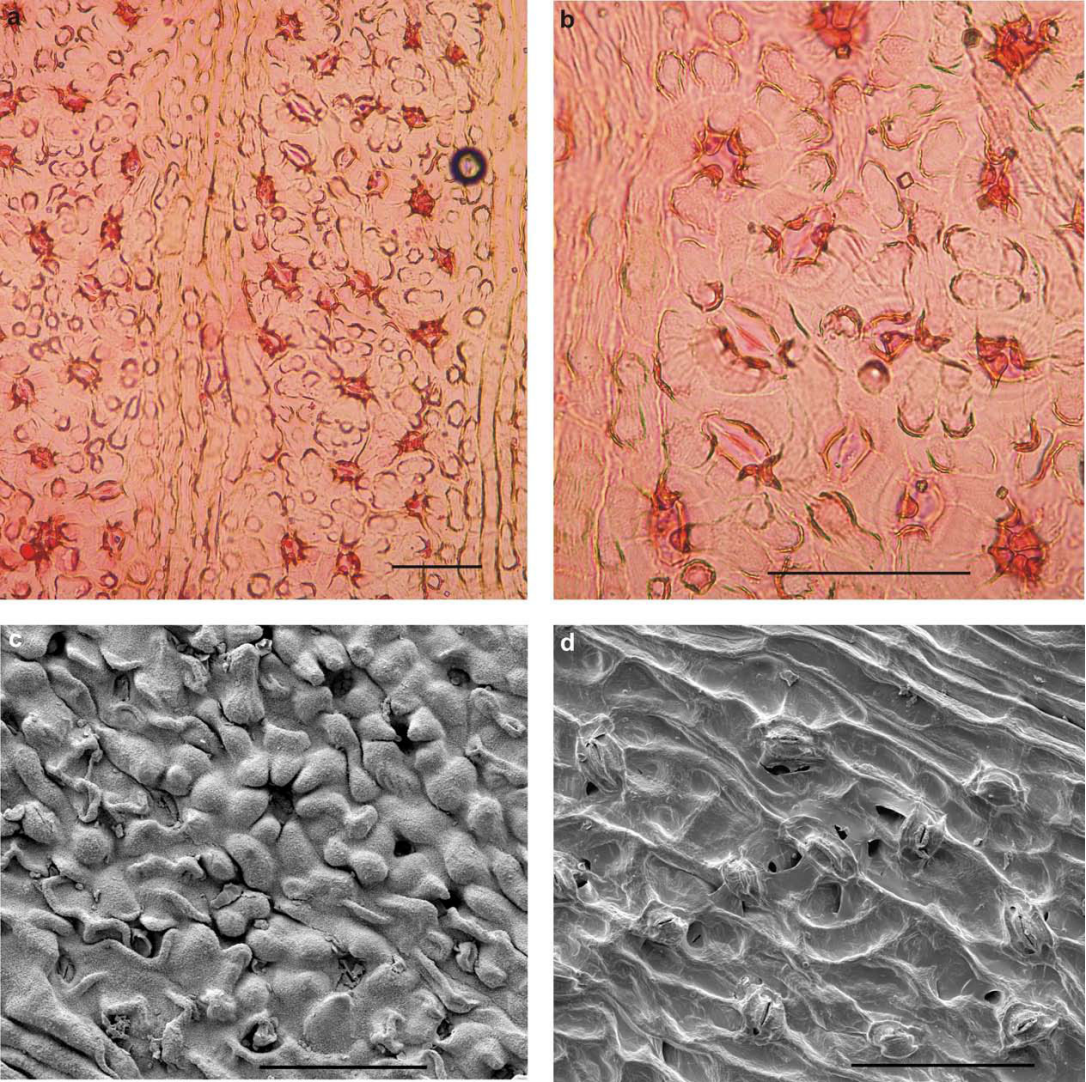
***Abaxial cuticles of the *O-ha-tsuki *leaf of *Ginkgo biloba**. a: Abaxial cuticle of the lamina with two costal rows without stomata and two intercostal areas with numerous stomatal complexes (inventory number Herbarium BOZ PVASC15174). Scale bar 100 μm. b: Detail of the abaxial cuticle of the lamina. Note the variable thickening and cutinization of the papillae of the subsidiary cells. Scale bar 100 μm. c: Outside of the abaxial cuticle of the lamina with numerous stomatal complexes, which are variable in the numbers and shape of the papillae (SEM). Scale bar 100 μm. d: Inner side of the abaxial cuticle of the lamina with several stomatal complexes (SEM). Scale bar 100 μm.

The abaxial cuticle of the extant *O-ha-tsuki *leaf of *Ginkgo biloba *(figure 9a-d) shows the distinct pattern typical of *Ginkgo *leaves: narrow costal rows without stomata and broader areas in between (intercostal fields) with numerous stomata. The narrow costal fields are 100-125 μm wide and consist of three to five rows of longitudinal, mostly rectangular cells. The anticlinal walls are indistinct but seem to be straight to very slightly undulating. Papillae occur not on every cell, but often. The intercostal areas are much broader than the costal areas, at least 200 μm or more. Numerous stomata are randomly oriented and are irregularly distributed as well as the papillae. The anticlinal walls between the stomata are indistinct, but mainly straight or curved. The stomata are enclosed by 5-6 (-7) subsidiary cells with prominent marginal papillae; sometimes the papillae are less strong or not observable. The stomata are elongated, their pores do not exceed 40 μm in length, and the inner walls of the guard-cells are slightly thickened in the middle part. The aperture is about 20-25 μm. The outside surface of the whole abaxial cuticle shows a fine granulated structure.

**Figure 10:**
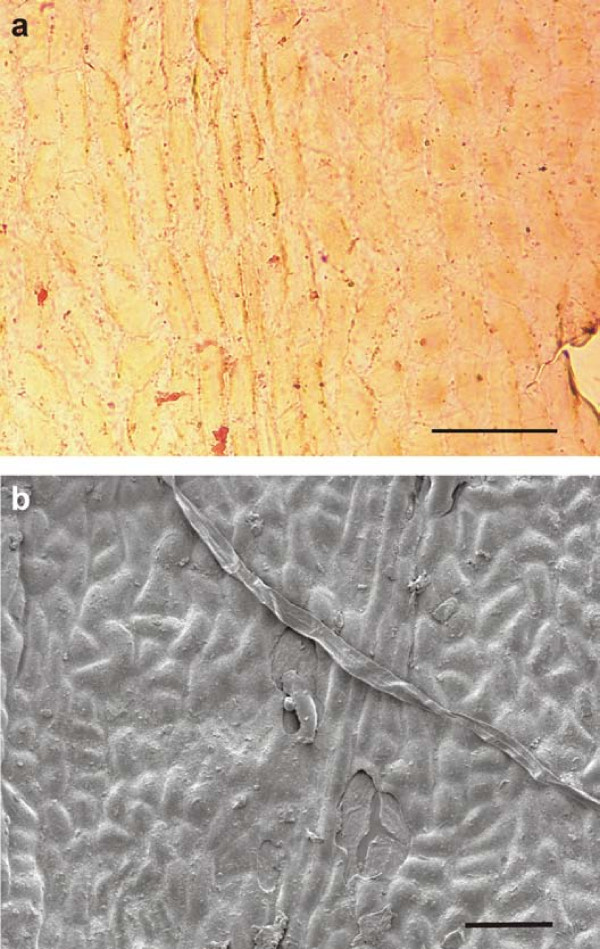
***Adaxial cuticles of the *O-ha-tsuki *leaf of *Ginkgo biloba**. a: The adaxial thin cuticle of the lamina. Scale bar 100 μm. b: Outside of the adaxial cuticle of the lamina with one costal area and two intercostal areas. The pit in the centre of the picture might indicate a stoma (SEM). Scale bar 100 μm.

The adaxial cuticle of the leaf (figure 10a, b) is very thin and without or almost devoid of stomata and papillae. The cuticle consists of narrow rows of mainly rectangular to narrow elongated cells (about 90 μm long and 35 mm wide) (costal areas) and broader areas (intercostal areas) with wider cells of rectangle elongated shape (about 70 μm long and 35 μm wide). The anticlinal walls are mainly undulating.

**Figure 11:**
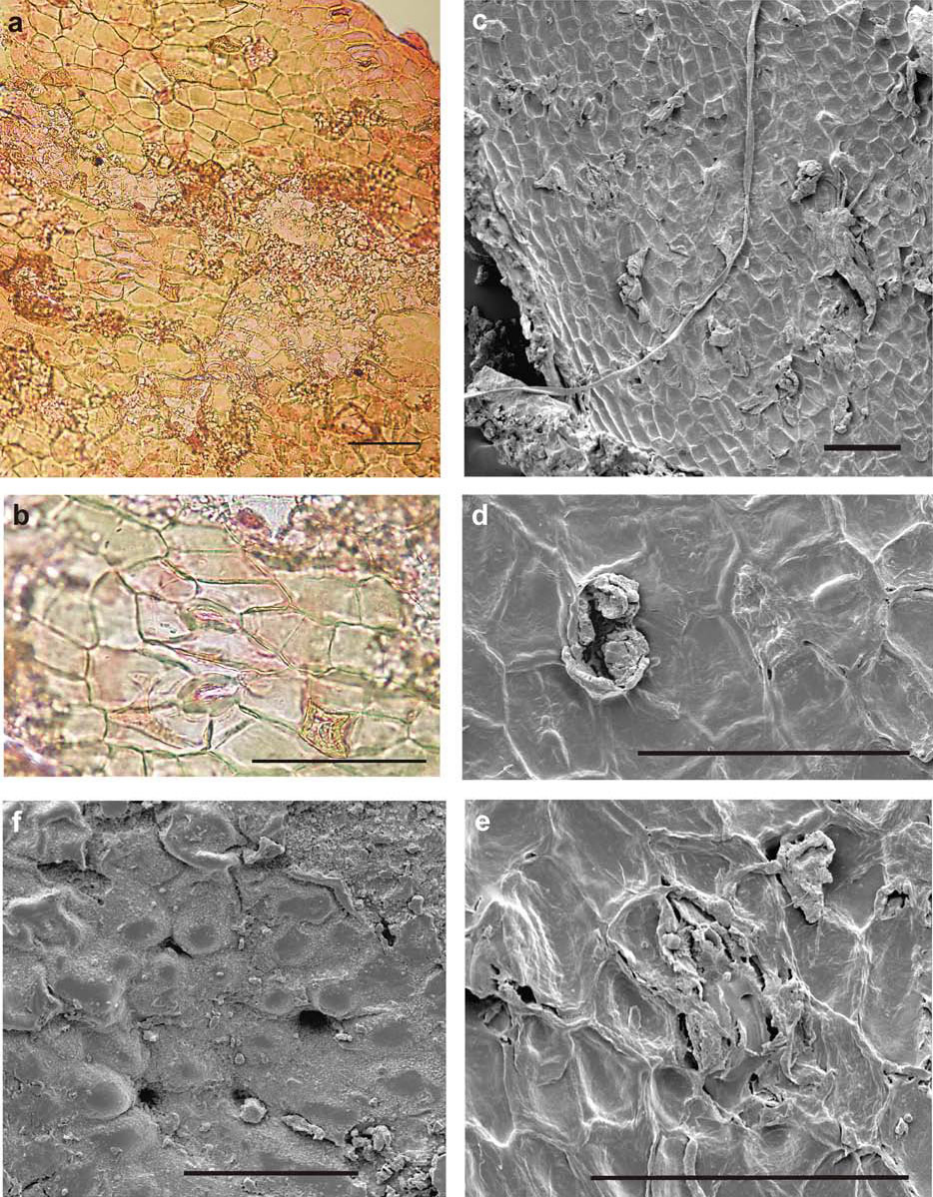
***Cuticles of the *O-ha-tsuki *ovule of *Ginkgo biloba**. a: Overview of the ovule cuticle with some stomatal complexes. Scale bar 100 μm. b: Two stomatal complexes next to each other in detail and one star-like idioblast above the scale bar. Scale bar 100 μm. c: Inner side of the ovule cuticle with some idioblasts and stomatal complexes (SEM). Scale bar 100 μm. d: An idioblast in detail (SEM). Scale bar 50 μm. e: One stomatal complex (SEM). Scale bar 100 μm. f: Outside of the ovule cuticle with papillae. Some are arranged around pits, indicating stomata (SEM). Scale bar 100 μm.

The cuticle of the *O-ha-tsuki *ovule epidermis (figure 11a-f) consists of isodiametric and polygonal to rectangular cells, which are irregularly arranged. The anticlinal walls are straight to curved. Papillae have not been observed with the LM. The cell diameter varies from 25 to 45 μm. The stomatal complex is built by 5-6 subsidiary cells and two small guard cells. The subsidiary cells are partly irregular in shape and up to 60 μm in diameter; their periclinal walls sometimes seem striate (LM), the inner anticlinal walls are distinctly thickened. The stomata are about 20-25 μm long. Idioblasts are also regularly observable. The outside surface of the ovule cuticle is mainly smooth, but some papillae surrounding a putative stomatal pore are observable (figure 11 f: SEM).

#### Further specimens of Sphenobaiera leaves associated with seeds from the Bletterbach

**Figure 12:**
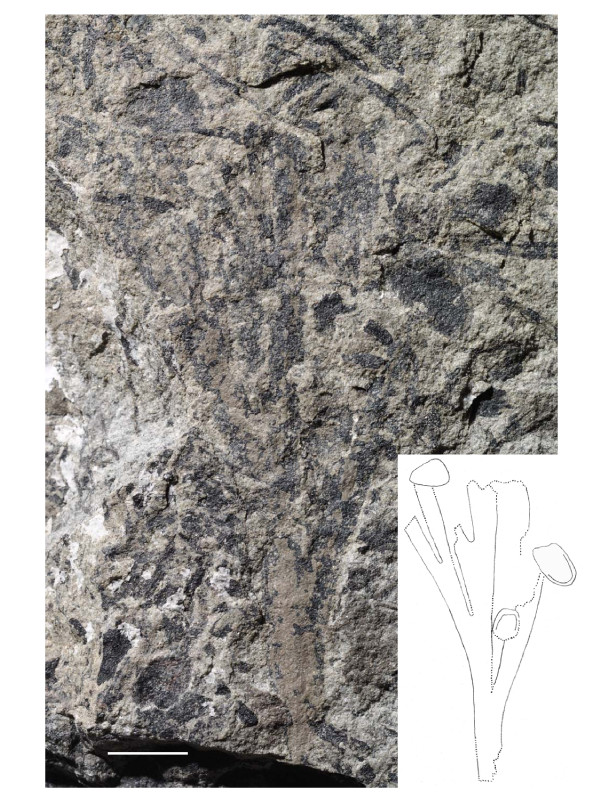
**Second specimen of a ginkgophyte leaf with putatively attached ovules from the Upper Permian of the Bletterbach**. Morphology of the specimen and drawing (inventory number PAL-1369). The specimen is preserved within a sediment layer with several plant remains. Scale bar 10 mm.

A second specimen of a *Sphenobaiera *leaf which shows putatively attached seeds is shown in figure 12. This leaf has multiple segments and three seeds are preserved in positions apical to the respective leaf segments. In total, seven specimens of *Sphenobaiera *leaves with putatively attached or associated seeds have been found at the Bletterbach locality (others not shown) where at least the position of the seeds suggests the attachment to the leaves, but the individual preservation does not allow proving organic connection in these cases.

## Discussion

The systematic position of the leaves with putatively attached seeds from the Bletterbach could principally be supposed among the cycads, the pteridosperms, or the ginkgophytes. Cycadophytes, however, are characterized by pinnately (or rarely bipinnately) compound leaves, most unlike the Bletterbach fossils. Among pteridosperms the character of interest "leaf-borne seeds" is found, but leaflets are mostly not dichotomous. There are Y-shaped fronds which, in case of forms with entire margin (non-pinnate) like the Triassic *Dicoidium dutoitii*, show similarity in gross morphology ([14], figure 15.20) to once-dichotomous leaves like PAL-1368, but not to the multiple-dichotomous forms of *Sphenobaiera *from the Bletterbach (figure 12). Most importantly, these leaves possess central veins (the rachis). In contrast, in well preserved *Sphenobaiera *specimens from the Bletterbach dichotomous/parallel venation can be observed; and a prominent midvein should be observable in PAL-1368 but is absent. The Permian pteridosperm *Dichophyllum flabellifera *([14], figure 15.57) possesses divided pinnules similar to *Sphenobaiera *leaves, but pinnules are tongue-shaped and have midveins. Based on the described morphology an affiliation of PAL-1368 to the pteridosperms can be excluded. The leaves with putatively attached seeds can be identified as belonging to the ginkgophyte genus *Sphenobaiera*.

The results of the cuticle studies on PAL-1368 also support the ginkgophyte origin of the fossil leaf and seed. The differences of leaf and seed cuticles are comparable to these differences in modern *Ginkgo biloba *or its *O-ha-tsuki *form. The cuticle structures of the fossil seed and those of extant *O-ha-tsuki *ovules show similar isodiametric to rectangular cell shapes and idioblasts in both cases, which may represent aborted stomata, trichome bases or glands. For *Ginkgo biloba *leaves Florin [24] mentions the rare occurrence of trichomes. With the SEM it is visible, that the cuticle is covered with wax. The indistinct parts of the LM pictures are caused by that. Two tubular structures (SEM) assumed to consist of wax were observed, this has also been described for *Ginkgo biloba *and other gymnosperms [25]. In contrast to the impression from the LM picture, the stomata are also surrounded by papillae on the surface. The fossil seed cuticle differs from the extant one only by the distinctly thicker anticlinal walls and the thickened subsidiary cells.

Cuticle structures especially of Permian *Sphenobaiera *leaves have only been described by Florin [24] from the Lower Permian of France (*Sphenobaiera raymondi*) and by Schweitzer [26] from the Upper Permian of Germany (*Sphenobaiera digitata*). The cuticle of the *Sphenobaiera raymondi *specimen is not well preserved. It seems to be composed mainly of isodiametric cells instead of longitudinal ones, possesses straight cell walls, and stomatal complexes are specified as haplocheilic with narrowing of the stomatal pore. *Sphenobaiera digitata *cuticles are described as amphistomatic, with a cuticle showing longitudinally oriented cells, rows of stomata towards the center of the lamina and less regular cells and stomata distribution towards the margin. Papillae are present only on subsidiary cells. Stomatal complexes are described as dicyclic, but seem to be mainly monocyclic as is shown in the drawing given in [26].

Fossil ginkgophyte leaves are often amphistomatic with rare stomata in the adaxial cuticle, but hypostomatic leaves have also been described [24] and have been found also among common *Ginkgo biloba *leaves as well as varying stomata abundances in apical and basal leaf parts. Furthermore, stomata index for *Ginkgo biloba *was reported to be inversely correlated with CO_2 _concentration [27]. Varying stomata abundance is also described for a fossil ginkgophyte [28]. Hypostomatic ginkgophyte leaves are represented by a *Sphenobaiera *species from the Triassic Molteno Formation in South Africa [[23], p. 133] and by *Baiera *cf. *furcata *from the Middle Jurassic of China [29]. From the Jurassic Yorkshire Flora [21]*Sphenobaiera ophioglossum *shows similar cuticle structures (cell types of costal and intercostal fields, stomatal complexes, presence of papillae) [[21], figure 17 D], but is designated as the adaxial cuticle. The abaxial cuticle of *Sphenobaiera schenckii *from the Triassic of South Africa [23] is seemingly identical, but its adaxial cuticle shows abundant stomatal complexes (not present in the adaxial cuticle of the Bletterbach specimen). Several *Sphenobaiera *leaves from the Bletterbach have to be studied in future for detailed taxonomic comparisons and specific identification of these leaves.

With respect to the cuticle of the fossil seed depicted here, the cells of the outer cuticle of the integument of *Ginkgo yimaensis *ovules [7, plate 3, figure four] are very similar and the description also concurs. One distinct difference is the size of the stomatal complex, which is much larger (150-175 × 7.5-17.5 (-35) μm) and less circular than in the specimen from Bletterbach. The cuticle of the outer integument of the ovules, described for *Yimaia recurva *(associated with leaves of *Baiera hallei*) [30] differs by the rounded corners of the cells, the larger stomatal complexes and the unspecialized subsidiary cells. The cuticle of the Jurassic ovule *Yimaia qinghaiensis *[31] closely resembles that of the seed from the Bletterbach with respect to its general reticulate structure formed by irregular-polygonal and irregularly arranged cells, the absence of papillae on these cells, and its scattered stomatal complexes; even if any of these characters is not uncommon among gymnosperms. The Cretaceous ovuliferous organ *Nehvizdyella bipartita *[11] possesses the same cuticular characteristics as the Jurassic ovule *Yimaia qinghaiensis *and as the cuticle of the *Sphenobaiera *seed from Bletterbach. *Ginkgo ginkgoidea *(Tralau) Yang, Friis et Zhou [31] from the Jurassic of Sweden shows also alike cuticle structures.

Ontogenetic developmental aberrations can resemble primitive phylogenetic states of organs, commonly called "atavisms". Ideally, the aberrant character closely resembles the primitive character, which can either be known by the fossil record of the group, or by identification of the primitive state of the character by comparison with living representatives of sister groups and outgroups for the clade. For the aberrant *O-ha-tsuki *leaves of *Ginkgo biloba *the underlying genetics is completely unknown. An alternative interpretation of the phenomenon to atavism would be its description as a case of ectopic organ development, which offers no mechanistic explanation. Rothwell expected fossil occurrence of ginkgophyte brachyblasts (spur shoots) with sporophylls like the *O-ha-tsuki *leaves [[32], p.101]. Anderson and Anderson [17] also led attention to such *"*anomalous strobili with leafy expansions" (= *O-ha-tsuki *form) of *Ginkgo biloba *comparing them with their Triassic *Avatia bifurcata *fructification, even without a distinct laminar structure of the considered fossil. However, it can not be excluded that these Triassic fructifications represent rare aberrant forms.

*Sphenobaiera *represents a heterogeneous group of leaf fossils with wide stratigraphical distribution. The leaf morphogenus comprises dichotomous and wedge-shaped leaves, which do not possess a petiole like extant *Ginkgo *and the fossil leaf morphogenera *Ginkgo*, *Baiera *and *Ginkgoites*. Most species of *Sphenobaiera *are thought to represent ginkgophytes [14].

**Figure 13:**
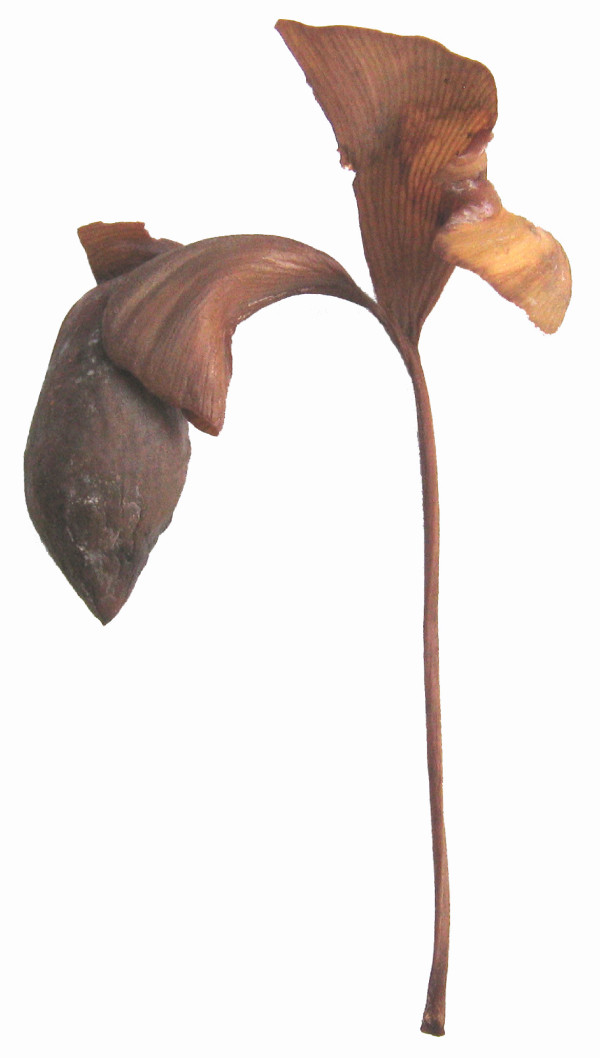
**O-ha-tsuki *leaf of *Ginkgo biloba *with well developed ovule***. Courtesy H Okada (inventory number Herbarium BOZ PVASC15174).

The *Sphenobaiera *leaves with putatively attached seeds described here from the Bletterbach locality could possibly represent cases of ectopic organ development, present for an unknown reason. However, this seems unlikely since rare aberrations would not be expected in smaller collections of fossils. The specimen PAL-1368 reveals striking similarity especially with *O-ha-tsuki *leaves with few or only one well developed ovule (figure 13). *O-ha-tsuki *leaves with multiple ovule formation often only show malformed ovules.

Zhou and Zheng [12] suggested an evolutionary scheme starting with Jurassic *Ginkgo yimaensis *with multiple small ovules and multiple divided vegetative leaves, subsequent reduction of ovule number and number of leaf segments in Cretaceous *Ginkgo apodes *and Cainozoic *Ginkgo adiantoides*, ending with *Ginkgo biloba *with predominantly only one ovule per stalk and bilobate to undivided vegetative leaves ("reduction hypothesis", "peramorphosis"). Given that the interpretation of the presented Permian fossils from Bletterbach as sporophylls is correct; these would represent an ancestral state of female ginkgophyte sporophylls before reduction of the sporophyll lamina had taken place, predating the reconstructed evolutionary series.

## Conclusions

The *Sphenobaiera *fossils with seemingly attached seeds from the Upper Permian of the Bletterbach are hypothetically interpreted as ancestral ginkgophyte sporophylls with laminar structure, as would be expected from aberrant *O-ha-tsuki *leaves with seeds of extant *Ginkgo biloba*. A formal description or affiliation of the fossils at the species level will be provided together with the one for the numerous vegetative *Sphenobaiera *leaves and with the other floral elements. Ongoing excavations at the Bletterbach locality, at other Permian localities, but also screening of museum collections could provide more and possibly better preserved ginkgophyte leaves with ovules/seeds. Especially specimens with completely preserved vascular systems would be highly desirable for comparison with vegetative and fertile organs of extant *Ginkgo biloba*.

## Methods

### Excavation of the fossils and deposition

The fossils were recovered during excavations in the years 2003-2009. The figured specimens are kept in the palaeontological collection of the Museum of Nature South Tyrol (Bozen/Bolzano, figure three: no. PAL-1368, figure twelve: no. PAL-1369).

### Cuticular analysis

Small pieces of organic material were removed from the fossil with a scalpel, rinsed in water and treated with a drop of 10% HF until all sediment particles were dissolved. The material was rinsed in water and incubated in conc. HNO_3 _with KClO_3 _(Schulze's Reagent) until the organic material became transparent. The material was rinsed with water, briefly treated with 5% KOH, rinsed with water, and transferred into glycerol for study and storage. The samples of the *O-ha-tsuki *leaf and seed were taken from one specimen, from the middle part of the lower and upper half of the leaf and seed and directly put into Schulze's Reagent. One piece was studied with the LM, the other with the SEM. The *O-ha-tsuki *leaf sample for the LM has been stained with safranin. The cuticles were studied with a Nikon eclipse 80i light microscope (LM), the pictures had been taken with a Samsung digimax V70. For the scanning electron microscope FEI (SEM), the macerated wet cuticles were transferred to the SEM-stub with a carbon adhesive tape, where they dried. One piece of the fossil leaf cuticle (slide one) was removed from the glycerol, washed with water and ethanol and then picked onto carbon tape on the SEM-stub. During the drying process a rather distinct shrinkage of the cuticles has been observed, which results in partly smaller cell sizes of the SEM cuticles. Both, fossil and extant leaf cuticles are very thin and fragile; the cuticles of the seeds are thick. These thick cuticles tended to roll in and a strong mechanical pressure was necessary to get a nearly flat cuticle. The pictures were slightly adjusted with Adobe Photoshop 7.0 in brightness, contrast and frame.

### Drawing of fossils

Drawings of fossils were performed using a binocular to discriminate biological structures from those produced by local destruction.

### *O-ha-tsuki *specimens

The specimens have been conserved in aqueous formaldehyde solution and are kept in the Herbarium of the Museum of Nature South Tyrol BOZ (PVASC15174).

## Authors' contributions

BM and TF discovered the new site with plant macroremains in the Bletterbach gorge. RB, BM, EK and TF excavated the macroremains from the site, prepared and studied the material. EK organized the excavations and took care for the museum collection of the Bletterbach material. BM performed the cuticular analyses. RB, BM, and TF studied literature on other ginkgophytes. TF and BM wrote the manuscript. All authors read and approved the final manuscript.
